# Transcriptome Analysis of B Cell Immune Functions in Periodontitis: Mucosal Tissue Responses to the Oral Microbiome in Aging

**DOI:** 10.3389/fimmu.2016.00272

**Published:** 2016-07-18

**Authors:** Jeffrey L. Ebersole, Sreenatha S. Kirakodu, M. John Novak, Luis Orraca, Janis Gonzalez Martinez, Larry L. Cunningham, Mark V. Thomas, Arnold Stromberg, Subramanya N. Pandruvada, Octavio A. Gonzalez

**Affiliations:** ^1^Center for Oral Health Research, College of Dentistry, University of Kentucky, Lexington, KY, USA; ^2^Division of Periodontics, College of Dentistry, University of Kentucky, Lexington, KY, USA; ^3^Caribbean Primate Research Center, Sabana Seca, PR, USA; ^4^Division of Oral and Maxillofacial Surgery, College of Dentistry, University of Kentucky, Lexington, KY, USA; ^5^Department of Statistics, College of Arts and Sciences, University of Kentucky, Lexington, KY, USA; ^6^Division of Orthodontics, College of Dentistry, University of Kentucky, Lexington, KY, USA

**Keywords:** B cells, periodontitis, aging, gingival tissues, non-human primates

## Abstract

Evidence has shown activation of T and B cells in gingival tissues in experimental models and in humans diagnosed with periodontitis. The results of this adaptive immune response are noted both locally and systemically with antigenic specificity for an array of oral bacteria, including periodontopathic species, e.g., *Porphyromonas gingivalis* and *Aggregatibacter actinomycetemcomitans*. It has been recognized through epidemiological studies and clinical observations that the prevalence of periodontitis increases with age. This report describes our studies evaluating gingival tissue transcriptomes in humans and specifically exploiting the use of a non-human primate model of naturally occurring periodontitis to delineate gingival mucosal tissue gene expression profiles focusing on cells/genes critical for the development of humoral adaptive immune responses. Patterns of B cell and plasmacyte genes were altered in aging healthy gingival tissues. Substantial increases in a large number of genes reflecting antigen-dependent activation, B cell activation, B cell proliferation, and B cell differentiation/maturation were observed in periodontitis in adults and aged animals. Finally, evaluation of the relationship of these gene expression patterns with those of various tissue destructive molecules (MMP2, MMP9, CTSK, TNFα, and RANKL) showed a greater frequency of positive correlations in healthy tissues versus periodontitis tissues, with only MMP9 correlations similar between the two tissue types. These results are consistent with B cell response activities in healthy tissues potentially contributing to muting the effects of the tissue destructive biomolecules, whereas with periodontitis this relationship is adversely affected and enabling a progression of tissue destructive events.

## Introduction

“Negative, age-related changes in our innate and adaptive immune systems are known collectively as immunosenescence. A lifetime of stress on our bodies is thought to contribute to immunosenescence. Radiation, chemical exposure, and exposure to certain diseases can also speed up the deterioration of the immune system.”^1^ This general concept conveyed by the National Institute of Aging has been supported by a wealth of epidemiological data, which has laid a foundation to better understand the age-related changes in immune functioning that facilitate increased development of an array of diseases later in life. Aging is a degenerative process with hallmarks of chronic inflammation and oxidative stress-related mitochondrial damage that contribute to the initiation of other deleterious processes within cells, including epigenetic modification of gene expression that can affect normal cellular functions (1). Alterations in both innate and adaptive immunity have been universally observed in aging populations and have led to the birth of such terms as “immune-aging” or “immunosenescence” to reflect the deteriorating nature of the immune system (2). Studies have shown that increases in the incidence of inflammation, autoimmunity, cancer, and susceptibility to infections in aging individuals coincide with a significant decline in host immunity and can negatively affect the efficacy of vaccinations in the elderly (3, 4).

Age-related reductions in the adaptive immune response are often accompanied by the presentation of a chronic low-grade inflammatory state, termed “inflammaging,” which can be influenced both by genetic and environmental factors and contributes to the individual variation observed within the population (5). Under normal circumstances, immunoactivation is a host defense response that evolved to protect humans against numerous pathogens. However, recent studies are showing that the coincident loss of normal innate and adaptive immune response capacity with aging, combined with low-grade chronic inflammation, work together to effectively alter immunocompetence and promote the pathogenesis of a diverse number of diseases (6). These findings suggest that “unhealthy” aging is driven in part by the dysregulation of immunoactivation (7). Alterations in positive and negative feedback signaling with aging appear to occur with all type of cells involved in immune responses; from short-lived neutrophils to long-lived T lymphocytes and macrophages. Recently, a review by Boraschi et al. (8) summarized several possible causes of immunosenescence and provided some strategies for counteracting this decline of immune responsiveness with aging.

Animal models, frequently focusing on the use of various genetically unique strains of laboratory mice, have been employed to advance our knowledge of aging and will continue to be useful tools. These types of studies can address many of the basic questions pertaining to the molecular regulation and/or dysregulation of the immune response and increased inflammation that accompanies aging. More recent studies have utilized these approaches in rodents to examine the impact of aging on periodontitis, and generally supported an increase in disease with aging (9–11). However, there are many fundamental differences in the basic immunology of mice and humans, particularly with respect to the microbiome composition, characteristics of immune cell transcriptional regulators, and receptors utilized for engaging pathogens and commensal bacteria, as well as the types of environmental stressors. Moreover, the rodent model has generally focused on the potential role of individual molecules within complex pathways in attempting to explain susceptibility or resistance to disease (12–16). These studies generally do not address the biology of the system that will be necessary to clearly define the multivariate changes that occur with healthy and diseased aging (2). Thus, Franceschi et al. (17) have proposed a “network theory of aging” emphasizing a broad reduction in the ability of the host response systems to cope with the array of challenges in concert with a progressing intrinsic proinflammatory environment that accompanies the aging process. These concepts emphasize a remaining need for sophisticated and detailed documentation of the human or human-like models of aging and immunosenescence (17).

While substantial emphasis has been placed on studies of innate immune and inflammatory responses in chronic periodontitis over the decades (18), extensive findings have described local and systemic antibody responses that (i) react with certain bacteria (19–21), (ii) increase with periodontitis, and (iii) are altered by treatment (22). Examination of the gingival tissue transcriptome in various studies have either “cast a broad net” to identify the multitude of differences between health and disease (23–27) or focused on innate immune response molecules (28–31). This article describes our ongoing studies focusing on the use of a non-human primate model of periodontitis to explore the response environment in the gingival mucosal tissues. We spotlight in this report on cellular markers of humoral adaptive immunity related to aging and chronic inflammatory disease of the oral mucosa and alveolar bone.

Various discussions regarding the role of adaptive immunity, and particularly humoral immune responses in the development of, or protection from, periodontitis have occurred over the decades, with some consistency in findings regarding antibody levels increased with disease (20, 22); however, severe generalized disease may show some decreased antibody levels (32–35). Treatment of periodontal disease usually associated with early increases but long-term decreases with successful therapy in antibody specific for oral bacteria (36–40). Animals models have also demonstrated the potential for antigen-specific T and B cells to exacerbate local disease (41–43), whereas active immunization has demonstrated protection from induced disease (16, 44–47).

## Materials and Methods

### Human Gingival Samples

Gingival tissue samples were obtained from three healthy and four periodontitis patients following Institutional Review Board approval and patient consent to utilize discarded tissues from standard of care surgical therapy for periodontitis and impacted third molar removal. Healthy tissues were uninflamed obtained from third molar extractions, patients aged 25–36 years, 66% females. The periodontitis samples were obtained *via* surgical therapy from sites with pocket depth ≥6 mm and radiographic evidence of alveolar bone loss, aged 32–51 years, 50% females. The samples were obtained and placed into chilled buffer. Total RNA was isolated from each gingival tissue specimen using TRIzol reagent following the protocol recommended by the manufacturer (Invitrogen, Indianapolis, IN, USA). The extracted RNA was cleaned up using the Qiagen RNeasy mini kit (Qiagen, Valencia, CA, USA) and quantified using spectrophotometric analysis. RNA from each sample was reverse transcribed and hybridized to the GeneChip^®^ HT Human Genome U133 Plus 2.0 Array (Affymetrix, Santa Clara, CA, USA) similar to methods we have described previously. Probe arrays were scanned using GeneChip Scanner 3000 for high resolution scanning and GeneChip Operating Software MAS 5.0.

### Non-Human Primate Experimental Design

Rhesus monkeys (*Macaca mulatta*) (*n* = 34; 14 females and 20 males) housed at the Caribbean Primate Research Center (CPRC) at Sabana Seca, PR, USA, were used in these studies. A cross-sectional study of healthy and naturally occurring periodontitis (48) animals were categorized as: young healthy (≤3 years; *n* = 5), adolescent healthy (3–7 years; *n* = 5), adults (12–16 years) healthy (*n* = 7) and periodontitis (*n* = 5), and aged (18–23 years) healthy (*n* = 6) and periodontitis (*n* = 6). The non-human primates were typically fed a 20% protein, 5% fat, and 10% fiber commercial monkey diet (diet 8773, Teklad NIB primate diet modified: Harlan Teklad). The diet was supplemented with fruits and vegetables, and water was provided *ad libitum* in an enclosed corral setting.

A protocol approved by the Institutional Animal Care and Use Committee (IACUC) of the University of Puerto Rico, enabled anesthetized animals to be examined for clinical measures of periodontal health, including probing pocket depth (PPD), and bleeding on probing (BOP) as we have described previously (49). Naturally occurring periodontitis sites were defined as PPD ≥4 mm and BOP ≥1.

### Microarray Analyses

A buccal gingival sample from either healthy or periodontitis-affected tissue from the premolar/molar maxillary region of each animal was taken using a standard gingivectomy technique, and maintained frozen in RNAlater solution. Total RNA was isolated from each gingival tissue using a standard procedure as we have described, and tissue RNA samples submitted to the microarray core to assess RNA quality analyze the transcriptome using the GeneChip^®^ Rhesus Macaque Genome Array (Affymetrix) (48, 50). Individual samples were used for gene expression analyses.

### Statistical Analyses

Normalization of values across the chips was accomplished through signal intensity standardization across each chip using Affymetrix PLIER algorithm. The arrays contained matched and mismatched pairs allowing the MAS 5 algorithm to be used. For each gene, we first determined differences in expression across the groups using ANOVA (Version 9.3, SAS Inc., Cary, NC, USA). The healthy aged tissues were then compared with the other age groups using a *t*-test and accepting a *p*-value ≤0.05 for significance. Because of the cost of these types of non-human primate experiments and availability of primates of the various ages, we did not have sufficient samples to identify if the relationship between age and gene expression could be treated using a linear model; thus, the subjects were classified, and ANOVA was used for analysis. The choice of Least Significant Difference for multiple comparisons (ANOVA followed by *t*-tests) provided maximum power given our necessarily small sample sizes. We determined a correlation with aging in healthy tissues using a Spearman Rank correlation analysis that was fit to the gene expression by age. A *p*-value ≤0.05 was used to evaluate the significance of the correlation. Volcano plots were prepared to visualize outlier gene expression profiles in the aging animals compared with the other age group (51). The heat maps with hierarchical clusters were prepared using MeV_4_9_0 (open source genomic analysis software).^2^ These data have been uploaded into the ArrayExpress data base^3^ under accession number: E-MTAB-1977.

## Results

### Gingival Transcriptome Alterations in Aging and Periodontitis

**Figure 1:**
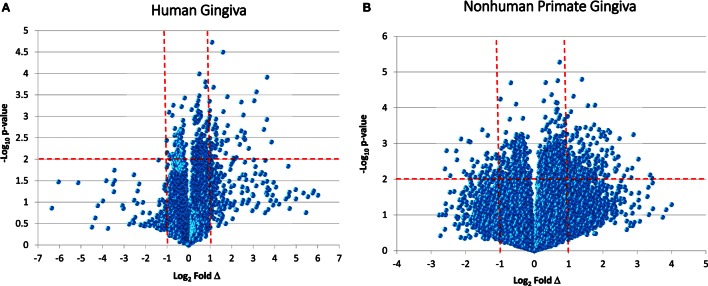
**Volcano plots of gene expression in human gingival tissues (A) and non-human primate gingival tissues (B) comparing levels in periodontitis tissues from healthy tissues**. Human samples (*n* = 3 healthy; *n* = 4 periodontitis) and non-human primate samples [*n* = 13 healthy; *n* = 11 periodontitis (adults and aged)] were tested. The graphs displays a point for the expression of each gene in periodontitis versus healthy tissues plotted as log_2_ fold difference (Δ) and −log_10_ *p*-value significance versus healthy tissues. The vertical red dashed lines signify a fold difference in expression at twofold and the horizontal red dashed line signifies a significant difference of *p* < 0.01.

While reports have documented the end result of adaptive immune cell functions, i.e., antibody, rather sparse information is available regarding the cellular changes that occur in the periodontitis gingival tissues with respect to the adaptive immune response. Figure 1 provides volcano plots depicting the characteristics of gene expression differences in healthy and naturally occurring periodontitis tissues from humans and non-human primates. This profile provides a depiction of the magnitude of change in each gene (i.e., Fold Δ) and the level of significant difference (i.e., *p*-value) in each gene comparing periodontitis to healthy tissues. Table 1 summarizes the identification of the top 50 upregulated genes in periodontitis gingival tissues from humans and non-human primates compared with healthy gingiva. The interesting aspect of these findings was that of the top 50 upregulated expressed genes in periodontitis tissues from humans and the non-human primates 40–60% were representative of genes that would affect the adaptive immune response including antigen presentation and processing, B and T cell regulation, and regulation of immunoglobulin characteristics. These findings support an active role in the complex gingival environment of established periodontitis lesions for the adaptive immune response. Whether this response profile is a hallmark for the development and expression of a protective immune response remains unclear in human disease (20, 22, 52). Furthermore, the effects of aging on these patterns of responses remain understudied.

**Table 1:** **Identification of the top 50 upregulated genes in periodontitis versus healthy gingival tissues related to adaptive immune responses**.

Human		Non-human primate	

Gene ID	r(i)	Gene ID	r(i)
V-DXP4-JH6c	24.7	IGHV-III VH26	10.29
IGKVJ	14.39	IGHV-II SESS	8.87
IGVH1	12.43	VPREB2	8.64
V-DK4-JH4b	12.33	IGKV-1 HK102	8.49
C-D-JH6	11.84	IGKV-11 RPMI6410	7.69
IGL	11.22	CD179b	7.57
IGK-3D-15	8.6	IGKV-1 HK101	6.17
IGLV2-23*01	8.2	KLRC1	6.16
MUC5AC	8.08	MMP3	5.81
NHPHL7	6.9	IGHV4-1	5.75
SCYB6	6.16	IGLV-I BL2	5.73
GRO1	6.1	ZLG	5.49
IGLJ3	6.03	PIP	5.41
TMEM156	5.89	IGLV-II MGC	5.2
IGHG3	5.78	IGHA2	5.19
PABL	5.42	FAM123A	5
KYNU	5.4	CLDN8	4.75
IL8	5.15	IGLV-III LOI	4.73
UBD	4.71	IGH	4.44
VNN1	4.62	IGJ	4.39
SLU7	4.19	ADCYAP1	4.37
IGKVJ	4.11	TVB2 CTL-L17	4.35
IGVH4	3.9	IGHV-II ARH-77	4.26
CPD	3.79	IGHV	4.26
PLAC8	3.51	POU2AF1	4.25
IGKVJC	3.49	APOE	4.23
SOD2	3.45	MCART6	4.15
IGK	3.34	IL19	4.13
IGLP3	3.27	hmRNPA1	4.12
CASP3	3.11	CXCL1	4.05
MS4A2	3.06	CCL20	4.05
CD20	3	IGLL1	4.04
MDM4	2.89	KLRF1	3.87
PLAT	2.87	COX6C	3.75
GLDC	2.81	HLA-DOB	3.7
CEACAM1	2.71	FAM30A	3.64
YES1	2.62	FOSB	3.57
PTH	2.53	CENPT	3.53
XBP1	2.49	SPATA4	3.52
FMO2	2.47	FAM46C	3.49
SOD2	2.45	IGL	3.43
PSME4	2.44	SPAST	3.39
SLC1A1	2.41	IGHG1	3.39
C20orf67	2.38	TNNI2	3.38
HS3ST1	2.38	LILRA1	3.38
CLCA4	2.34	IGLV-II BUR	3.38
GPR65	2.34	BANK1	3.37
PHLDA1	2.33	IGHV-I V35	3.36
HSP13A	2.16	IRF4	3.27
SLP76	2.15	TACI	3.27
IGA1C	2.13	IGK	3.26

*Genes highlighted in yellow associated with adaptive immune responses*.

### B Cell- and Plasmacyte-Associated Transcriptome in Aging and Periodontitis

**Figure 2:**
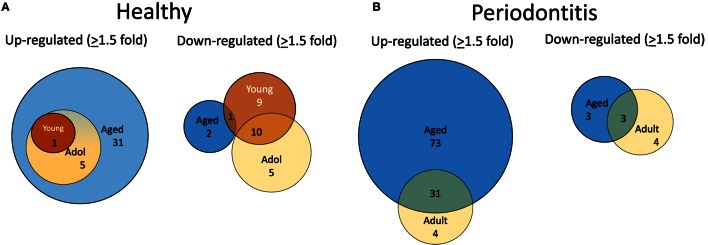
**Venn diagram depicting altered gene expression in healthy tissues (A) and periodontitis tissues (B) compared with levels in healthy adult tissues**. Gene numbers denote fold differences by ≥1.5-fold. Naturally occurring periodontitis does not occur in younger age groups.

To evaluate alterations in B cell development and maturation in the gingival tissue, we documented expression of 190 genes that are associated with B cell and plasmacyte development and functions. We also categorized the B cell genes into those related to B cell activation, proliferation, and differentiation, as well as antigen-stimulated activation of these cells. The plasmacyte genes were related to general cellular expression and those suggested to be altered in early or mature plasmacytes. Figure 2A is a diagrammatic representation of altered gene expression profiles in healthy gingival tissues from young, adolescent, and aged animals compared with levels in healthy adult tissues. The results show that 31 genes are expressed at higher levels in the healthy aged tissues, whereas only 1 and 5 were elevated in young and adolescent healthy gingiva compared with healthy adult tissues. The figure also demonstrates that all gene changes in the young and adolescent overlapped with the changes in aged tissues. In contrast, only 2 of this gene set were downregulated in healthy aging tissues, whereas 19 were significantly decreased in young and 15 in adolescent tissues with approximately 50% overlapping. Figure 2B presents an overview of the prevalence of genes that were up- or downregulated in periodontitis in the adult or aged animals (naturally occurring periodontitis does not present in the younger animal groups). The results showed that two times more genes were upregulated in aged periodontitis tissues compared with the adult group. Approximately 90% of the elevated genes in adult periodontitis tissues were also elevated in the aging tissues. In contrast, very few genes were downregulated with periodontitis in either age group, with only about 1/2 of these genes overlapping in the two age groups.

We then examined the characteristics of these altered genes related to B cell functions. Table 2 summarizes these alterations in healthy tissues of each of the age groups compared with levels in healthy adult tissues. The majority of gene expression changes in the healthy tissues were associated with antigen-dependent activation and B cell differentiation/maturation processes (26.3 and 37.9%, respectively). As was noted in the Venn diagrams, the majority of the gene changes were elevations in aged healthy tissues across all functional groups.

**Table 2:** **Alterations in gene expression in healthy gingival tissues related to gene functional categories**.

Gene functions	Total genes	Altered gene expression						
		Total	Young		Adolescent		Aged	

		↑ or ↓	↑	↓	↑	↓	↑	↓
Antigen-dependent activation	38	10	0	6	1	3	8	0
B cell activation	18	4	0	1	0	0	3	3
B cell proliferation	33	6	0	2	0	2	5	0
Differentiation/maturation	58	22	1	8	4	9	18	1
Plasmacyte	25	2	0	1	0	2	1	0
Multiple functions	17	3	0	2	0	2	1	0

*↑ or ↓ denotes gene expression that was up- or downregulated compared with healthy adult tissues*.

**Figure 3:**
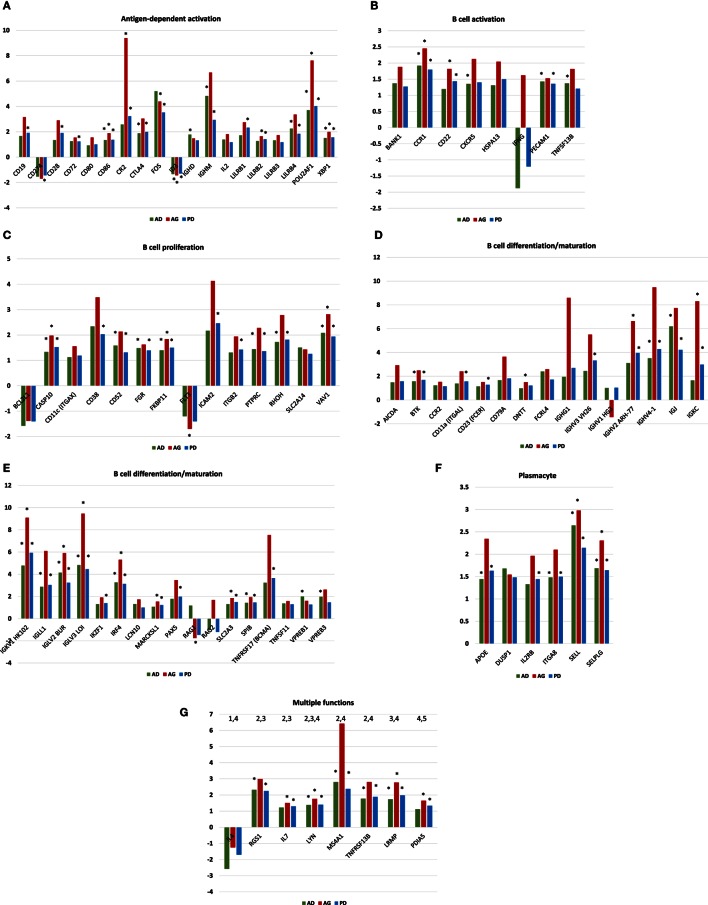
**(A–G)** Gene expression profiles of periodontitis from adult (AD), aged (AG), and overall periodontitis (PD) group compared with levels in healthy adult tissues for each of the various B cell/plasmacyte functional categories. The bars denote the mean fold difference of disease from health. Only those genes with a fold difference ≥1.5 or ≤−1.5 in any of the three comparisons were included in the graph (21/38). The asterisk defines comparisons that were significantly different at least at *p* < 0.05.

Figures 3A–G provide the results of gene expression changes in the various functional categories related to periodontitis in adults and aged animals, as well as a summary of all periodontitis samples. With the antigen-dependent activation genes, 21 out of 38 were altered with periodontitis, generally being increased in both age groups, and most often with fold increases greatest in the aged group. Only CD274 (programed death-ligand 1 with a major role in suppressing the immune system through a signal that inhibits TCR-mediated activation of IL-2 production and T cell proliferation) and ID3 (DNA-binding protein inhibitor ID-3 is a transcription inhibitor) were decreased with periodontitis. B cell activation genes were altered (8/18) with periodontitis, again showing higher fold increases in the aged tissues. Approximately 14/33 B cell proliferation-related genes were altered with periodontitis in both adults and aged animals. Of these, only BCL2L1 (potent inhibitor of cell death by inhibiting activation of caspases) and FLT3 (Fms-like tyrosine kinase 3 or CD135 is important or lymphocyte development) were consistently decreased. Examination of the B cell differentiation/maturation genes showed 32/58 were altered in periodontitis (Figures 3D,E). Again the highest fold increases were in the aged tissue samples. Interestingly, one-third of these genes were associated with genes related to immunoglobulin heavy and light change rearrangement and production. Only 6/25 plasmacyte-related genes were altered with periodontitis with all being increased in both adult and aged animals. Finally, a group of genes with multiple functions in humoral immune responses are presented (Figure 3G) with 8/17 being altered, and only IL4 being consistently decreased in adult and aged periodontitis tissues.

**Figure 4:**
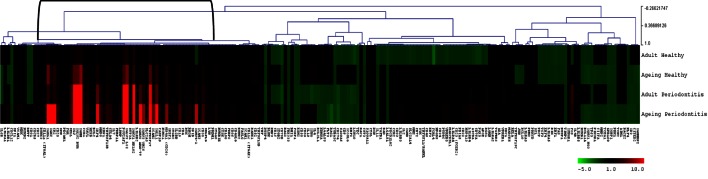
**Heatmap of fold differences in gene expression in adult and aged healthy and periodontitis tissues compared with response levels in healthy gingival tissues from a group of 10 animals 3–7 years of age (approximately 11- to 25-year-old humans)**. The bracket identifies a cluster of genes that were highly expressed in adult and aged tissues, particularly with periodontitis.

Figure 4 provides a heatmap of the clustering of the B cell/plasmacyte genes in healthy and periodontitis samples from adults and aged animals. The results provide a visualization of a cluster of genes that are clearly elevated in expression in healthy aged tissues and then increase substantially in periodontitis or more specifically, only in aged periodontitis tissues. Table 3 provides a summary of these gene expression changes identified with their primary gene functions. Six of the 26 gene alterations were related to antigen-dependent stimulation of the B cells, generally increasing sequentially from adult to aged healthy tissues and then continuing increased with adult to aged periodontitis tissues. The majority of changes were associated with differentiation/maturation functions, particularly related to immunoglobulin diversity. The changes were noticeable in that the fold-increase approximately doubled from adult to aged healthy, and then the changes were threefold to sevenfold increased comparing healthy adult to periodontitis or healthy aged to periodontitis.

**Table 3:** **Gene expression differences in adult and aged healthy and periodontitis tissues related to average gene response patterns in healthy tissue samples from animals 3–7 years of age (correlates with 11- to 25-year-old human)**.

	Fold difference				
	
Gene ID	Adult H	Aged H	Adult PD	Aged PD	Fxn. Category^a^
CCR1	1.2	1.8	2.4	3.1	2
CD19	1.7	2.5	2.8	5.2	1
CD28	1.5	1.8	2.0	4.3	1
CD20 (MS4A1)	1.1	3.3	3.2	7.3	2.4
CR2 (CD21)	1.4	3.7	3.5	12.8	1
CTLA4	1.2	1.8	2.3	3.8	1
FOS	0.8	1.5	4.4	3.7	1
ICAM2	1.1	1.4	1.3	1.7	3
IGHG1	2.2	6.3	4.2	18.5	4
IGHM	2.5	7.4	12.2	16.9	4
IGHV2 ARH-77	1.6	2.4	5.1	10.8	4
IGHV3 VH26	1.3	1.8	3.2	7.1	4
IGHV4-1	0.9	1.8	3.2	8.5	4
IGJ	2.4	5.5	14.9	18.6	4
IGKC	1.3	2.9	2.1	10.4	4
IGKV1 HK102	3.1	4.1	14.6	27.9	4
IGLV2 BUR	2.6	5.5	10.9	15.5	4
IGLV3 LOI	3.0	6.7	14.6	28.7	4
IGLL1	1.9	3.7	5.4	11.5	4
IRF4	1.8	3.2	6.0	9.7	4
LRMP	1.2	1.5	2.1	3.3	3.4
POU2AF1	2.8	5.1	10.4	21.4	1
SELL	1.7	2.7	4.4	5.0	5
TNFRSF13B	1.4	2.1	2.5	4.0	2.4
TNFRSF17	3.0	5.8	9.6	22.3	4
VAV1	1.4	2.1	2.9	4.0	3

*^a^Functional categories denoted as 1 – antigen-dependent activation; 2 – cell activation; 3 – cell proliferation; 4 – differentiation/maturation; 5 – plasmacyte; 6 – multiple functions*.

**Figure 5:**
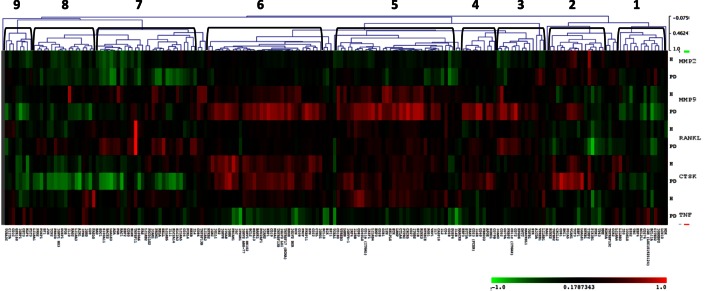
**Heatmap depicting the distribution of correlations of B cell/plasmacyte genes with tissue destructive genes (MMP2, MMP9, CTSK, TNF, and RANKL) in healthy gingival tissues (H, *n* = 23) and periodontitis tissues (PD, *n* = 11) of adult and aged animals**. The graph displays differences in gene expression in the adult and aged tissues compared with control levels displayed in a group of 10 healthy animals from 3 to 7 years of age. Green denotes negative correlation and red denotes positive correlation with the expression of the tissue destructive genes. The brackets signify the nine gene clusters created based on similarity indices of 0.4–0.5.

Since host-induced tissue destruction is a feature of the chronic inflammation of periodontitis, we explored the relationship between gingival expression of the B cell/plasmacyte group of genes and expression of genes associated with periodontal tissue destruction during health and periodontal disease irrespective of age. Included in these tissue destructive responses were evaluation of MMP2 (72 kDa type IV collagenase or gelatinase A active in extracellular matrix degradation and effects on integrins), MMP9 (92 kDa type IV collagenase or gelatinase B, a regulatory factor in neutrophil migration and functions), CTSK (a lysosomal cysteine protease involved in bone remodeling and resorption, can catabolize elastin, collagen, and gelatin), TNF (tumor necrosis factor alpha or cachexin is a cell signaling protein involved in systemic inflammation and contributes to the acute phase response with cell cytotoxic capabilities), and RANKL (receptor activator of nuclear factor kappa-B ligand also TNFSF11 is a principal factor in osteoimmunology linking the immune system and bone biology, as well as an apoptosis regulatory gene). The heatmap shows the correlations in either healthy or periodontitis samples of the B cell genes with the expression of tissue destructive genes (Figure 5) and provides an overview of the correlation of the B cell/plasmacyte genes with the five tissue destructive genes in healthy and periodontitis tissues (adult and aged combined). Cluster 1 contained 15 genes showing a positive correlation with TNFα and negative correlation with MMP9, primarily in the periodontitis tissues. Cluster 2 genes (*n* = 12) were positively correlated with the expression of MMP2 and CTSK in both health and periodontitis, as well as with MMP9 only in periodontitis. Cluster 3 was a subset with 14 genes that negatively correlated with MMP2 in healthy samples and positively correlated with MMP9, RANKL, and CTSK in only the periodontitis samples. Cluster 4 contained 10 genes that were positively correlated with both MMP9 and CTSK in periodontitis. Cluster 5 genes (*n* = 34) showed a positive correlation with MMP9, RANKL, and CTSK in both healthy and periodontitis tissues, and a positive correlation with TNFα, but only in the healthy tissues. Cluster 6 contained 34 genes with strong positive correlations with MMP9 and CTSK in both healthy and periodontitis tissues, and a negative correlation with TNFα in periodontitis. Cluster 7 grouped 28 genes with positive correlations to RANKL only in periodontitis tissues, and strong negative correlations to CTSK in periodontitis and MMP2 in both healthy and periodontitis samples. Cluster 8 genes (*n* = 18) were similar to cluster 7 with negative correlations to MMP2 and CTSK in periodontitis and health, as well as negative correlations with MMP9 in periodontitis tissues. Finally, cluster 9 genes (*n* = 8) were similar to expression patterns for cluster 8, except for positive correlations with TNFα in periodontitis.

Table 4 summarizes the distribution of functional categories of the genes in each of these clusters and demonstrates some skewing of gene functions related to the various clusters of association with tissue destructive gene expression profiles. Clusters 1, 4, and 8 showed an expected general distribution across the functions. Cluster 3 showed an increased frequency of differentiation/maturation genes and decreased plasmacyte expression profiles. Clusters 4, 5, and 7 all exhibited increased proportions of the multiple function B cell genes. Cluster 6 was enriched for non-antigen-dependent B cell activation genes, whereas cluster 7 was depleted in this gene function. Finally, cluster 9, while containing a low number of genes was enriched for B cell proliferation genes, while being decreased for differentiation/maturation genes.

**Table 4:** **Functional categories of genes in the various clusters of response profiles related to expression of a set of tissue destructive genes (TDGs)**.

Cluster	TDG profile^a^	Gene functional category					
		Ag-dep. activation	Act.	Prolif.	Diff./Mat.	Plasmacyte	Multiple fxn
1	↓9; ↑T	4 (3)^b^	1 (1)	2 (3)	4 (5)	3 (2)	1 (1)
2	↑2; ↑C	**0 (2)**	1 (1)	3 (2)	4 (4)	2 (1)	2 (1)
	PD: ↑9						
3	PD: ↑9; ↑C; ↑R	4 (3)	2 (1)	2 (2)	**6 (4)**	**0 (2)**	0 (1)
	H: ↓2						
4	PD: ↑9; ↑C	1 (2)	0 (1)	0 (2)	4 (3)	2 (1)	**3 (1)**
5	↑9; ↑C; ↑R	8 (7)	3 (3)	6 (6)	**8 (11)**	4 (4)	**5 (2)**
	H: ↑T						
6	↑9; ↑C; ↓T	7 (7)	**6 (3)**	6 (6)	10 (11)	3 (4)	2 (2)
7	↓2; ↑R; ↓C	5 (6)	**1 (3)**	5 (5)	10 (9)	5 (4)	**4 (2)**
8	↓2; ↓C	5 (4)	2 (2)	2 (3)	5 (6)	3 (3)	1 (1)
	PD: ↓9						
9	↓C	3 (2)	0 (1)	**3 (1)**	**1 (3)**	1 (1)	0 (1)
	PD: ↓9; ↑T						
	H: ↓2						

*^a^TDG profile denotes ↑, positive correlation; ↓, negative correlation; 2 – MMP2; 9 – MMP9; C – CTSK; R – RANKL; T – TNFα. H denotes only in healthy tissues, and PD denotes only in periodontitis tissues*.

*^b^Numbers denote frequency of genes observed in individual clusters and number of genes expected based on total genes representing that function (parentheses)*.

*Those highlighted in bold indicate altered distribution*.

## Discussion

Dysregulation of multiple components of the immune system with aging contributes to increased prevalence and severity of infections and poses a challenge for prevention of infectious diseases *via* effective vaccination in aging populations. These dysfunctions have been described for adaptive immune B and T cells. Thus, aging individuals’ exhibit increased susceptibility to a number of inflammatory and degenerative pathologies. Included in this listing is an increased prevalence and severity of periodontitis, although the underlying causes remain poorly understood. The effects of aging on periodontal tissues have been suggested to be based on molecular changes in the array of cells of the periodontium, the combination of which is thought to intensify alveolar bone resorption in elderly individuals. These effects are considered to be reflective of (1) altered differentiation/proliferation of cells for uncoupling bone biological processes (osteoblasts, osteoclasts); (2) enhanced responses to the oral microbiota, modified by environmental stressors leading to the secretion of cytokines/chemokines involved in osseous resorption; and (3) systemic endocrine alterations related to host responses and physiologic/pathologic bone responses with aging (53–56). Hajishengallis (57) proposed that an impaired modulation of the innate immune and inflammatory capacity of the host could also be associated with aging-related periodontitis. Nevertheless, less attention has been placed on the potential role of aging-related adaptive immunity changes that could be contributing to higher incidence and severity of periodontitis with aging. In general, most of the evidence is supported by studies demonstrating that innate immune and adaptive immune cells isolated from aged individuals exhibit intrinsic defects that could predispose the elderly to dysregulated immune and inflammatory responses underpinning the exacerbated clinical features of disease with aging. Integrated approaches examining cellular biology, animal models, and human studies of aging should contribute to targeted molecular therapies that could mitigate the initiation and progression of periodontitis in aging and/or reverse the effects of aging on periodontitis as a chronic inflammatory disease.

The literature has clearly described the characteristics of the humoral adaptive immune response across gingival health toward various forms of periodontal disease with immunoglobulins (antibodies) of all isotypes generally present at low levels in gingival crevicular fluid from healthy sites, minimizing the potential for various hypersensitivity reactions that could contribute to local tissue destruction. There is also a wealth of literature supporting the existence of local specific antibody production by plasma cells present in inflamed tissues of the periodontal pocket because levels of local antibody can be significantly greater than those in the serum (19, 58, 59). However, the conundrum of existing data is why there appears to be a coincidence of chronic oral infection with accompanying periods of exacerbated disease and an active, often substantial specific local and systemic immune response in periodontitis (19, 22). At present, with our current level of knowledge, the true significance and function of the adaptive immune response under normal circumstances remains to be elucidated, let alone how aging specifically modulates the effectiveness of these responses.

Numerous alterations in B cells occur with aging (60–62). A critical aspect of B-lymphocytes is the heterogeneity of the stimulated mature plasmacytes differing in terms of the antigenic specificity of their resulting antibody-combining sites. Dunn-Walters and Ademokun (63) have suggested that one consequence of aging effects on the humoral immune system is an altered B-cell diversity; however, how this process occurs which remains unclear. Various studies have identified possible differences in lymphocyte subpopulations across the lifespan that has been proposed to contribute to the onset of age-related variations of the adaptive immune responses (64–66), such that the *in situ* environments in old age are considered to be (1) reduced in B-cell generation and (2) altered in the readout of the antibody repertoire. Riley (64) has suggested that in old age B cell development may progressively be diverted into a pre-BCR compromised pathway. While substantial literature is available over the last 40 years documenting levels of serum, gingival crevicular fluid, and salivary antibody to oral bacteria as they relate to periodontitis (19, 22), evidence is sparse regarding how these antibody responses are regulated with aging (22). However, early studies of these responses that implied an increase in antibody levels with age, really did not effectively distinguish the effect of aging directly on the immune response system versus response profiles that reflected increased extent and severity of periodontitis, e.g., oral infection, with aging (67).

The prevalence and severity of periodontitis, including missing teeth, increase significantly with age in various species of non-human primates (49, 68, 69), and the disease is associated with antibody responses in this animal model to a battery of oral bacteria, including periodontal pathogens (49). We have evaluated various aspects of cellular gene expression that would contribute to adaptive immune responses in gingival tissues using this non-human primate model of periodontitis, including antigen-presenting cells (70–72), characteristics of macrophage footprints in healthy and periodontitis gingival tissues from animals across their lifespan, as well as plasticity of the macrophage population in gingival tissues (73), and related to T cell phenotype/function during the initiation and progression of periodontitis related these to the expression of soft and bone tissue destruction genes (74).

This study extended these findings to the level of B cells and plasmacyte gene expression profiles with aging and periodontitis in this human-like animal disease model. While substantial research has developed focusing on innate immune and inflammatory molecular mechanisms of periodontitis (28, 57, 75, 76), we observed that within the upregulated transcriptome of both humans and non-human primates with periodontitis 40–60% of these genes were related to adaptive immune responses. These findings defined that, in fact, we still have a substantial gap in understanding the molecular events of adaptive immunity occurring in diseased gingiva that must play a critical role in the progression and/or resolution of the disease. Evaluation of transcriptomic footprints of B cells and plasmacytes provided a new view of patterns in healthy aging. First, a number of genes associated with the function of these cells were upregulated in healthy aged tissues, whereas an increased number of genes were significantly downregulated in healthy tissues from younger animals. These altered genes were primarily associated with what would be predicted to be an upregulation of B cell activation, proliferation, and differentiation/maturation processes occurring in the healthy aged tissues. Approximately 1/3 of the B cell/plasmacyte genes in healthy tissues were positively correlated with MMP9 expression levels across all of the functional classes of the B cell/plasmacyte genes, and approximately 1/2 of the genes in periodontitis were positively correlated with expression of this tissue altering enzyme. Results with CTSK responses showed a similar prevalence of positive correlations in healthy and periodontitis tissues, while a cluster of genes were negatively correlated with CTSK primarily in the periodontitis tissues. The patterns of responses related to RANKL showed that three of the clusters of B cell genes were generally positively correlated with RANKL, while only Cluster 5 was positively correlated in healthy tissues. High numbers of B cell/plasmacyte genes were also positively correlated with TNFα responses in the healthy tissues, focusing the most elevated levels in antigen-dependent activation genes. Summarizing these findings supported that increased B cell-related gene changes are observed in healthy aging tissues and that in healthy tissues (irrespective of age), generally B cell/plasmacyte-associated gene expression positively correlated with levels of expression of various tissue destructive biomolecules. An interpretation of these findings is that with healthy aging an increase in tissue destructive gene expression, as we have recently reported (77), is balanced by an active B cell-related response profile that enhances the likelihood of maintaining homeostasis of these mucosal tissues even with the overall impact of aging on the immune response system.

These findings contrasted with periodontitis tissues where upregulated genes in aging periodontitis tissues were often two times greater than the level expressed in adult periodontitis, with nearly complete overlap. Approximately 40–60% of the antigen-dependent activation, B cell activation, B cell proliferation, and B cell differentiation/maturation genes were altered with periodontitis, generally being increased in both adults and aged animals. Numerous genes related to immunoglobulin heavy and light change rearrangement and production (IGHG1, IGHM, IGHV2, IGHV3, IGHV4, IGJ, IGKC, IGKV1, IGLV2, IGLV3, and IGLL1) were substantially upregulated with the highest fold increases in aged animals with periodontitis. Additionally, complement receptor 2 (CR2, Epstein–Barr virus receptor, CD21), POU domain class 2-associating factor 1 (POU2AF1), and TNFRSF17 [B-cell maturation antigen (BCMA); tumor necrosis factor receptor superfamily member 17] were increased in aged periodontitis approximately10-fold compared with healthy adult tissues. CR2 is on mature B cells and engages with membrane associated CD19 and CD81 leading to a complex called the B cell coreceptor complex (78–80). CR2 binds to antigens through attached C3d when the B cell membrane IgM binds to specific antigen. This antigen-specific binding results in B cells having a substantially elevated response to the antigen and, thus, enabling the complement system to play a role in B-cell activation and maturation. POU2AF1 codes for a transcriptional coactivator, Obf1 (Bob.1) that specifically associates with either OCT1 (POU2F1) or OCT2 (POU2F2). It functions as a critical factor to accentuate OCT1- and OCT2-mediated promoter activation of immunoglobulin gene expression (81–83). It is essential, as part of the B cell receptor signaling pathway, for the response of B-cells to antigens and required for the formation of germinal centers (84, 85). TNFRSF17 is a member of the TNF receptor superfamily and binds B cell activating factor (BAFF; TNFSF13B), which is expressed in B lineage cells resulting in potent B cell activation, as well as NF-κB and MAPK8/JNK activation (86, 87). It is preferentially expressed on mature B cells and ligand engagement contributes to proliferation and differentiation of the B cells (78, 88). This receptor also binds to various TRAF family members (TNF receptor-associated factors) that interact with inhibitor-of-apoptosis proteins (IAPs) driving anti-apoptotic signals contributing to cell survival and proliferation (89, 90). These biomolecules and their associated pathways may provide novel targets for better understanding of the local adaptive immune response to the microbial insult that occurs with periodontitis.

Various discussions regarding the role of adaptive immunity, and particularly humoral immune responses in the development or protection from periodontitis have occurred over the decades, with some consistency in findings regarding antibody levels being increased with disease (22). However, a summation of the available literature from human studies regarding aging effects on humoral immune responses to oral microorganisms suggests rather minimal evidence to either support or refute that aging directly affects the capacity of individuals to mount an adaptive immune response to bacteria comprising the autochthonous oral microbiome including those associated with periodontitis. This report describes studies that emphasize a rather extensive alteration in the gingival environment with regard to B cell and plasmacyte activities in this non-human primate model of periodontitis and suggest changes that occur in healthy tissues, with aging, as well as extensive alterations associated with naturally occurring disease. How these responses are driven by changes in the microbiome of subgingival biofilms with disease and processes that may occur with adaptive immune responses to control the infection and reestablish homeostasis remains to be determined.

## Author Contributions

JE contributed to the experimental design and study activities, and also spearheaded the preparation of the manuscript and interpretation of the data. SK supported the preparation of the samples and interface with the core facility for microarray analysis. MN contributed to the experimental design and study activities, and also reviewed and contributed to content of the manuscript. LO supported the clinical aspects of evaluation and sample collection. JM provided support for conduct of the study and evaluation of the animals included in the experiment. LC provided human clinical specimens and reviewed the manuscript. MT provided human clinical specimens and contributed to discussion of the results. AS provided biostatistical support for the gene expression analyses. SP provided support for generation of the heatmap figures and interpretation of the results. OG responsible for the experimental design and oversight of the conduct of the study, and also contributed to content of the manuscript and review of the data.

## Conflict of Interest Statement

The authors declare that the research was conducted in the absence of any commercial or financial relationships that could be construed as a potential conflict of interest.

## Footnotes

^1^http://www.nia.nih.gov/health/publication/biology-aging/immune-system-can-your-immune-system-still-defend-you-you-age

^2^www.tm4.org

^3^www.ebi.ac.uk
