# Supplementary material for: Transcriptome Analysis of B Cell Immune Functions in Periodontitis: Mucosal Tissue Responses to the Oral Microbiome in Aging
Source: Front Immunol. 2016 Jul 18;7:272. doi: 10.3389/fimmu.2016.00272 (PMC4947588; doi:10.3389/fimmu.2016.00272)
Supplement: Supplementary file 1 [file table_1.pdf]

Supplementary Table S1: Gene expression targets

Probe ID	Gene ID	Gene Descriptions	Cell type	Functional Category
Antigen-dependent activation				
MmugDNA.32422.1.S1_at	CD180	CD180 molecule	B	1
MmugDNA.18254.1.S1_at	CD19	CD19 molecule	B	1
MmugDNA.14306.1.S1_at	CD1C	CD1c molecule	B	1
MmugDNA.20774.1.S1_at	CD274	CD274 molecule	B	1
MmugDNA.19231.1.S1_at	CD28	CD28 molecule	B	1
MmuSTS.3490.1.S1_at	CD72	CD72 molecule	B	1
MmugDNA.37827.1.S1_at	CD79B	CD79b molecule, immunoglobulin-associated beta	B	1
MmugDNA.29916.1.S1_at	CD80	CD80 molecule	B	1
MmugDNA.6430.1.S1_at	CD83	CD83 molecule	B	1
MmugDNA.32699.1.S1_at	CD86	CD86 molecule	B	1
MmugDNA.19832.1.S1_at	CIITA	class II, major histocompatibility complex, transactivator	B	1
MmuSTS.3942.1.S1_at	CR2	complement component receptor 2	B	1
MmugDNA.25496.1.S1_at	CTLA4	cytotoxic T-lymphocyte-associated protein 4	B	1
MmugDNA.5901.1.S1_at	FAS	Fas cell surface death receptor	B	1
MmugDNA.8857.1.S1_at	FASLG	Fas ligand (TNF superfamily, member 6)	B	1
MmugDNA.27012.1.S1_at	FKBP2	FK506 binding protein 2, 13kDa	P	1
MmugDNA.38565.1.S1_at	FOS	FBJ murine osteosarcoma viral oncogene homolog	B	1
MmuSTS.165.1.S1_at	ID3	inhibitor of DNA binding 3	B	1
MmugDNA.20492.1.S1_at	IGBP1	immunoglobulin (CD79A) binding protein 1	B	1
MmugDNA.2488.1.S1_at	IGHD	immunoglobulin heavy constant delta	B	1
MmuSTS.4350.1.S1_at	IGHM	immunoglobulin heavy constant mu	B	1
MmunewRS.438.1.S1_at	IL2	interleukin 2	B	1
MmugDNA.42882.1.S1_x_at	SPN	sialophorin	B,P	1
MmuSTS.4526.1.S1_at	SYK	spleen tyrosine kinase	B	1
MmuSTS.4672.1.S1_at	TNF	tumor necrosis factor	B	1
MmuSTS.1841.1.S1_at	XBP1	X-box binding protein 1	B,P	1
MmugDNA.35906.1.S1_at	CD40	CD40 molecule, TNF receptor superfamily member 5	B	1,3
MmugDNA.19086.1.S1_at	IL10	interleukin 10	B	1,3,4
MmugDNA.20386.1.S1_at	IL4	interleukin 4	B	1,4
MmuSTS.4100.1.S1_at	TEC	tec protein tyrosine kinase	B	1,4
B-cell activation				
MmugDNA.20995.1.S1_at	CCR1	chemokine (C-C motif) receptor 1	B	2
MmuSTS.3487.1.S1_at	CD22	CD22 molecule	B	2
MmugDNA.12740.1.S1_at	CXCR5	chemokine (C-X-C motif) receptor 5	B	2
MmugDNA.23725.1.S1_at	HSPA13	heat shock protein 70kDa family, member 13	B,P	2
MmugDNA.21955.1.S1_at	ICAM1	intercellular adhesion molecule 1	B	2

MmugDNA.34534.1.S1_at	ICAM3	intercellular adhesion molecule 3	B	2
MmugDNA.7551.1.S1_at	ICOSLG	inducible T-cell co-stimulator ligand	B	2
MmuSTS.173.1.S1_at	IFNG	interferon, gamma	B	2
MmugDNA.21956.1.S1_at	IFNGR1	interferon gamma receptor 1	B	2
MmugDNA.13394.1.S1_at	LILRA1	leukocyte immunoglobulin-like receptor, subfamily A, member 1	B	2
MmuSTS.2816.1.S1_at	LILRA2	leukocyte immunoglobulin-like receptor, subfamily A, member 2	B	2
MmugDNA.13391.1.S1_at	LILRA3	leukocyte immunoglobulin-like receptor, subfamily A, member 3	B	2
MmuSTS.215.1.S1_at	LILRA4	leukocyte immunoglobulin-like receptor, subfamily A, member 4	B	2
MmugDNA.31251.1.S1_s_at	LILRA5	leukocyte immunoglobulin-like receptor, subfamily A, member 5	B	2
MmugDNA.15576.1.S1_at	LILRB1	leukocyte immunoglobulin-like receptor, subfamily B, member 1	B	2
MmugDNA.33060.1.S1_x_at	LILRB2	leukocyte immunoglobulin-like receptor, subfamily B, member 2	B	2
MmugDNA.13397.1.S1_x_at	LILRB3	leukocyte immunoglobulin-like receptor, subfamily B, member 3	B	2
MmugDNA.31218.1.S1_at	LILRB4	leukocyte immunoglobulin-like receptor, subfamily B, member 4	B	2
MmuSTS.4604.1.S1_at	LILRB5	leukocyte immunoglobulin-like receptor, subfamily B, member 5	B	2
MmuSTS.1025.1.S1_at	NFKBIE	nuclear factor of kappa light polypeptide gene enhancer in B-cells inhibitor, epsilon	B	2
MmugDNA.28182.1.S1_at	PECAM1	platelet/endothelial cell adhesion molecule 1	B,P	2
MmuSTS.3029.1.S1_at	PRCP	prolylcarboxypeptidase (angiotensinase C)	P	2
MmuSTS.2318.1.S1_at	PRKCD	protein kinase C, delta	B	2
MmugDNA.27833.1.S1_at	TGFB1	transforming growth factor, beta 1	B	2
MmugDNA.32051.1.S1_at	TGFBR2	transforming growth factor, beta receptor II (70/80kDa)	B	2
MmuSTS.4607.1.S1_at	TNFRSF13C	tumor necrosis factor receptor superfamily, member 13C	B	2
MmugDNA.635.1.S1_at	TNFSF13B	tumor necrosis factor (ligand) superfamily, member 13b	B	2
MmuSTS.686.1.S1_at	IL7	interleukin 7	B	2,3
MmuSTS.3131.1.S1_at	RGS1	regulator of G-protein signaling 1	B	2,3
MmugDNA.20554.1.S1_at	LYN	v-yes-1 oncogene homolog	B	2,3,4
MmugDNA.17218.1.S1_at	ADA	adenosine deaminase	B,P	2,4
MmugDNA.5951.1.S1_at	MS4A1 (CD20)	membrane-spanning 4-domains, subfamily A, member 1	B	2,4
MmugDNA.16923.1.S1_at	TNFRSF13B	tumor necrosis factor receptor superfamily, member 13B	B	2,4
Cellular proliferation				
MmugDNA.15422.1.S1_at	AIM1	absent in melanoma 1	B	3
MmuSTS.3462.1.S1_at	BAD	BCL2-associated agonist of cell death	B	3
MmugDNA.16559.1.S1_at	BANK1	B-cell scaffold protein with ankyrin repeats 1	B	3
MmugDNA.3082.1.S1_at	BCL10	B-cell CLL/lymphoma 10	B	3

MmugDNA.3186.1.S1_at	BCL11A	B-cell CLL/lymphoma 11A (zinc finger protein)	B	3
MmuSTS.3201.1.S1_at	BCL2	B-cell CLL/lymphoma 2	B	3
MmugDNA.18029.1.S1_at	BCL2L1	apoptosis regulator Bcl-2-like LOC707407	B	3
MmuSTS.3285.1.S1_at	CASP10	caspase 10, apoptosis-related cysteine peptidase	P	3
MmuSTS.2919.1.S1_s_at	CD11c (ITGAX)	integrin, alpha X (C3 receptor 4 subunit)	B	3
MmuSTS.1849.1.S1_at	CD38	CD38 molecule	B,P	3
MmugDNA.15084.1.S1_s_at	CD52	CD52 molecule	B	3
MmugDNA.14377.1.S1_s_at	CD58	CD58 molecule	B	3
MmugDNA.6428.1.S1_at	CD81	CD81 molecule	B	3
Mmu.7236.1.S1_s_at	CD9	CD9 molecule	B,P	3
MmugDNA.5215.1.S1_at	DUSP4	dual specificity phosphatase 4	B	3
MmuSTS.4458.1.S1_at	FGR	Gardner-Rasheed feline sarcoma viral (v-fgr) oncogene homolog	B	3
MmugDNA.13690.1.S1_at	FKBP11	FK506 binding protein 11, 19 kDa	B	3
MmugDNA.43558.1.S1_at	FLT3	fms-related tyrosine kinase 3	B	3
MmugDNA.17463.1.S1_at	GPR160	G protein-coupled receptor 160	B	3
MmugDNA.11374.1.S1_s_at	ICAM2	intercellular adhesion molecule 2	B,P	3
MmuSTS.2912.1.S1_at	IL4R	interleukin 4 receptor	B,P	3
MmuSTS.249.1.S1_at	ITGB2	integrin beta-2-like	B	3
MmuSTS.1430.1.S1_at	KLF4	Kruppel-like factor 4 (gut)	B	3
MmugDNA.26175.1.S1_s_at	LGALS1	lectin, galactoside-binding, soluble, 1	B	3
MmugDNA.8321.1.S1_at	MARCKS	myristoylated alanine-rich protein kinase C substrate	B	3
MmugDNA.42566.1.S1_at	NFKB2	nuclear factor of kappa light polypeptide gene enhancer in B-cells 2 (p49/p100)	B,MP	3
MmuSTS.3737.1.S1_at	PTPRC	protein tyrosine phosphatase, receptor type, C	B	3
MmuSTS.3377.1.S1_at	RELA	v-rel reticuloendotheliosis viral oncogene homolog A	B	3
MmuSTS.2853.1.S1_at	RHOH	ras homolog gene family, member H	B,P	3
MmugDNA.9398.1.S1_at	RUNX2	runt-related transcription factor 2	B	3
MmugDNA.25329.1.S1_at	SLC2A14	solute carrier family 2, member 14	B	3
MmugDNA.21307.1.S1_at	TIMP2	TIMP metallopeptidase inhibitor 2	P	3
MmugDNA.33493.1.S1_at	TP63	tumor protein p63	P	3
MmugDNA.24681.1.S1_at	VAV1	vav 1 guanine nucleotide exchange factor	B	3
MmuSTS.2884.1.S1_at	CD27	CD27 molecule	B,P	3,4
MmuSTS.4537.1.S1_at	LRMP	lymphoid-restricted membrane protein	B,P	3,4
MmuSTS.3011.1.S1_at	NFKB1	nuclear factor of kappa light polypeptide gene enhancer in B-cells 1	B	3,4
MmuSTS.3758.1.S1_at	RAC1	ras-related C3 botulinum toxin substrate 1	B	3,4
Differentiation and maturation				
MmugDNA.9644.1.S1_at	AICDA	activation-induced cytidine deaminase	B	4
MmugDNA.13724.1.S1_at	BACE2	beta-site APP-cleaving enzyme 2	B	4
MmuSTS.3524.1.S1_at	BIK	BCL2-interacting killer (apoptosis-inducing)	B,P	4

MmugDNA.34.1.S1_at	BLNK	B-cell linker	B	4
MmugDNA.29374.1.S1_at	BMI1	Similar to 60S ribosomal protein L32 LOC694196	B,P	4
MmuSTS.4534.1.S1_at	BTK	Bruton agammaglobulinemia tyrosine kinase	B	4
MmugDNA.36749.1.S1_at	CCR2	chemokine (C-C motif) receptor 2	P	4
MmugDNA.22748.1.S1_at	CD11a (ITGAL)	integrin, alpha L	B	4
MmugDNA.39285.1.S1_at	CD23 (FCER2)	Fc fragment of IgE, low affinity II, receptor for (CD23)	B	4
MmuSTS.3358.1.S1_at	CD34	CD34 molecule	B	4
MmugDNA.22843.1.S1_at	CD79A	CD79a molecule, immunoglobulin-associated alpha	B	4
MmugDNA.41966.1.S1_at	DNTT	DNA nucleotidylexotransferase, terminal	B	4
MmugDNA.35031.1.S1_at	EBF1	early B-cell factor 1	B	4
MmuSTS.217.1.S1_at	EBF2	early B-cell factor 2	B	4
MmugDNA.12797.1.S1_at	EBF3	early B-cell factor 3	B	4
MmugDNA.7540.1.S1_at	EBF4	early B-cell factor 4	B	4
MmugDNA.37796.1.S1_at	FBXO33	F-box protein 33	B	4
MmugDNA.41570.1.S1_at	FCRL4	Fc receptor-like 4	B	4
MmugDNA.31335.1.S1_at	FOXP1	forkhead box P1	B	4
MmuSTS.2022.1.S1_at	HPGDS	hematopoietic prostaglandin D synthase	B	4
Mmu.15620.1.S1_at	IGH	Ig heavy chain uncharacterized LOC101925124	B	4
MmugDNA.24969.1.S1_at	IGHG1	Ig heavy chain similar to hCG2042717	B	4
MmugDNA.7664.1.S1_at	IGHMBP2	immunoglobulin mu binding protein 2	B	4
MmugDNA.28431.1.S1_x_at	IGHV@	similar to immunoglobulin heavy locus LOC100291190	B	4
MmugDNA.21021.1.S1_at	IGHV3-VH26	immunoglobulin heavy chain variable region V-III region VH26	B	4
MmugDNA.17912.1.S1_at	IGHV1 HG3	similar to hCG1773549, LOC100133862	B	4
MmugDNA.14906.1.S1_s_at	IGHV2 ARH-77	similar to hCG1812074, LOC100293559	B	4
MmugDNA.11413.1.S1_s_at	IGHV4-1	similar to hCG2043206, LOC100291786	B	4
MmunewRS.816.1.S1_s_at	IGJ	immunoglobulin J polypeptide	B	4
MmugDNA.39296.1.S1_at	IGKC	similar to hCG26659 LOC100130100	B	4
MmugDNA.743.1.S1_at	IGLL1	immunoglobulin lambda-like polypeptide 1	B	4
MmugDNA.37168.1.S1_at	IKZF1	DNA-binding protein Ikaros-like	B	4
MmugDNA.23105.1.S1_s_at	IGLV2 BUR	similar to Ig lambda chain, LOC100293440	B	4
MmugDNA.38781.1.S1_at	IGLV3 LOI	similar to hCG2040021, LOC100293277	B	4
Mmu.15443.1.S1_x_at	IGKV1 HK102	similar to Ig kappa chain V-I region HK102 precursor LOC652493	B	4
MmugDNA.213.1.S1_at	IL11	interleukin 11	B	4
MmugDNA.34657.1.S1_at	IRF4	interferon regulatory factor 4	B,P	4
MmugDNA.26477.1.S1_at	JUN	jun proto-oncogene	B	4
MmugDNA.14389.1.S1_at	KDM5A	lysine (K)-specific demethylase 5A	B	4
MmugDNA.3736.1.S1_at	LCN10	lipocalin 10	B	4
MmugDNA.1162.1.S1_at	MARCKSL1	MARCKS-like 1	B,P	4
MmuSTS.2991.1.S1_at	MME	membrane metallo-endopeptidase	B	4

MmugDNA.38077.1.S1_at	PAX5	paired box 5	B	4
MmuSTS.1127.1.S1_at	PDCD1LG2	programmed cell death 1 ligand 2	B	4
MmugDNA.31087.1.S1_at	PIK3R1	phosphoinositide-3-kinase, regulatory subunit 1 (alpha)	B	4
MmugDNA.39211.1.S1_at	PRDM1	PR domain containing 1, with ZNF domain	B,P	4
MmugDNA.13476.1.S1_at	RAG1	recombination activating gene 1	B	4
MmuSTS.3102.1.S1_at	RAG2	recombination activating gene 2	B	4
MmugDNA.6347.1.S1_at	RUNX3	runt-related transcription factor 3	B	4
MmugDNA.17370.1.S1_s_at	S100A6	S100 calcium binding protein A6	P	4
MmugDNA.7683.1.S1_at	SF1	splicing factor 1	B	4
MmugDNA.19747.1.S1_at	SLC2A3	solute carrier family 2, member 3	B,P	4
MmugDNA.2786.1.S1_at	SPI1	spleen focus forming virus (SFFV) proviral integration oncogene	B	4
MmugDNA.31701.1.S1_at	SPIB	Spi-B transcription factor (Spi-1/PU.1 related)	B,P	4
MmugDNA.42036.1.S1_at	TCF3	transcription factor 3 (E2A immunoglobulin enhancer binding factor)	B	4
MmugDNA.5879.1.S1_at	TNFRSF17	tumor necrosis factor receptor superfamily, member 17	P	4
MmugDNA.14490.1.S1_at	TNFSF11	tumor necrosis factor (ligand) superfamily, member 11	B	4
MmuSTS.4727.1.S1_s_at	TUBA3C	tubulin, alpha 3c	B	4
MmugDNA.16332.1.S1_at	VPREB1	pre-B lymphocyte 1	B	4
MmuSTS.2905.1.S1_at	VPREB3	pre-B lymphocyte 3	B	4
MmuSTS.3145.1.S1_at	NOMO1	NODAL modulator 1	P	4,5
MmugDNA.14099.1.S1_at	PDIA5	protein disulfide isomerase family A, member 5	P	4,5
MmuSTS.2037.1.S1_at	PDK1	pyruvate dehydrogenase kinase, isozyme 1	P	4,5
Plasmacyte functions				
MmugDNA.31898.1.S1_s_at	APOE	apolipoprotein E	EP	5
MmuSTS.1868.1.S1_at	CITED2	cbp/p300-interacting transactivator 2-like	P	5
MmugDNA.32965.1.S1_at	DCN	decorin	P	5
MmugDNA.38506.1.S1_at	EMP3	epithelial membrane protein 3	P	5
MmuSTS.911.1.S1_at	EZH2	enhancer of zeste homolog 2 (Drosophila)	EP	5
MmugDNA.732.1.S1_at	HEXB	hexosaminidase B (beta polypeptide)	P	5
MmugDNA.20381.1.S1_at	IL2RB	interleukin 2 receptor, beta	B, EP	5
MmuSTS.682.1.S1_at	IL6R	interleukin 6 receptor	P	5
MmugDNA.32094.1.S1_at	LGALS3	lectin, galactoside-binding, soluble, 3	P	5
MmugDNA.2300.1.S1_at	PKM2	pyruvate kinase, muscle	P	5
MmuSTS.2359.1.S1_at	S100A11	S100 calcium binding protein A11	P	5
Mmu.2304.1.S1_at	SELL	selectin L	EP	5
MmugDNA.27663.1.S1_at	SELPLG	selectin P ligand	P	5
MmugDNA.21146.1.S1_at	STT3A	subunit of the oligosaccharyltransferase complex	P	5
MmugDNA.150.1.S1_at	TCEA1	transcription elongation factor A (SII), 1	P	5
MmuSTS.2229.1.S1_at	TCN2	transcobalamin II	P	5
MmuSTS.4697.1.S1_at	VCAM1	vascular cell adhesion molecule 1	EP	5

MmugDNA.35432.1.S1_at	CST3	cystatin C	MP	6
MmugDNA.35537.1.S1_at	CTSL	cathepsin L1-like	MP	6
MmugDNA.11572.1.S1_at	CXCL12	chemokine (C-X-C motif) ligand 12 (SDF1)	MP	6
MmuSTS.4085.1.S1_at	DUSP1	dual specificity phosphatase 1	MP	6
MmugDNA.21448.1.S1_at	ITGA8	integrin, alpha 8	MP	6
MmugDNA.14544.1.S1_at	LGMN	legumain	MP	6
MmugDNA.30644.1.S1_at	MDK	midkine-like	MP	6
MmugDNA.27367.1.S1_x_at	SDC1	syndecan 1	MP	6

Cell type denotes: B – B cell, P – plasmacyte, EP – early plasmacyte, MP – mature plasmacyte
Functional category denotes: 1 – antigen-dependent activation, 2 – B cell activation; 3 – B cell proliferaton, 4 – B cell differentiation and maturation, 5/6 – plasmacyte related genes
