# Supplementary figures and images for: Transcriptome Analysis of B Cell Immune Functions in Periodontitis: Mucosal Tissue Responses to the Oral Microbiome in Aging
Source: Front Immunol. 2016 Jul 18;7:272. doi: 10.3389/fimmu.2016.00272 (PMC4947588; doi:10.3389/fimmu.2016.00272)

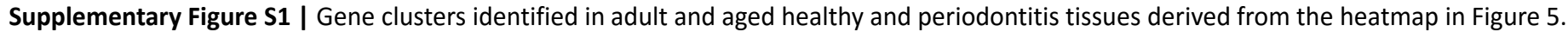

Supplement: Supplementary file 2 [file image_1.pdf]
